# Prevention of mother-to-child transmission of HIV guidelines: Nurses’ views at four primary healthcare facilities in the Limpopo Province

**DOI:** 10.4102/sajhivmed.v18i1.690

**Published:** 2017-06-28

**Authors:** Barbara A. Hanrahan, Adri Williams

**Affiliations:** 1 Department of Nursing, University of the Witwatersrand, South Africa; 2 Edupark, Polokwane, South Africa

## Abstract

**Background:**

When new guidelines for existing programmes are introduced, it is often the clinicians tasked with the execution of the guidelines who bear the brunt of the changes. Frequently their opinions are not sought. In this study, the researcher interviewed registered nurses working in the field of the prevention of mother-to-child transmission (PMTCT) of human immunodeficiency virus (HIV) to gain an understanding of their perspectives on the changes introduced to the guidelines. The guideline changes in 2014 were to move from the World Health Organization (WHO) Option B to Option B + which prescribes lifelong antiretroviral therapy (ART) for all HIV-positive pregnant women regardless of CD4 cell count.

**Objective:**

To determine what the registered nurses’ perspectives are on the PMTCT programme as implemented at four PHC facilities in the Limpopo Province.

**Method:**

For this qualitative investigation, a descriptive research design was implemented. The data were collected during semi-structured interviews with nurses from four primary healthcare facilities in the Limpopo Province of South Africa. Data were analysed using thematic analysis.

**Results:**

Challenges preventing effective implementation (e.g. increased workloads, viz. staff shortages; poor planning of training; equipment and medication shortages and long lead times; poor patient education) were identified.

**Conclusion:**

In spite of the successes of the PMTCT programme, considerable challenges still prevail; lack of patient education, poor facilities management and staff shortages could potentially influence the implementation of the PMTCT guidelines negatively.

## Introduction

### Background and literature review

Frequently, policy is determined by committees removed and distant from the actual sites of implementation, that is, a top-down approach. On the other hand, policy may be determined by the eventual beneficiaries, that is, a bottom-up approach. However, Bhuyan, Jorgensen and Sharma^1^ recommend including more role players, that is, ‘moving away from top-down or bottom-up dichotomies to a centrist approach emphasising how actors from different institutional contexts influence what gets implemented’. In the context of this research, the focus was on inputs gleaned from the perceptions of registered nurses (RNs) in the community-based health service.

The study was conducted on the implementation of the South African prevention of mother-to-child transmission (PMTCT) of human immunodeficiency virus (HIV) guidelines. The focus was on the evaluation of the implementation of the PMTCT guidelines, from the perspective of RNs within the context of primary healthcare (PHC) facilities.

The provision of antiretroviral therapy (ART) is an important part of the strategy to manage HIV infection. The guideline changes were announced in July 2014, published in December 2014 and implemented in January 2015.^2,3^ These changes were authorised for implementation for specific groups, for example, pregnant and breastfeeding women; infants and early adolescents; and late adolescents and young adults. The changes to the PMTCT programme prescribe that all HIV-positive women who are pregnant, breastfeeding or who are within one year postnatal are immediately initiated on lifelong ART – regardless of their CD4 cell count.

ART is often initiated by a registered or PHC nurse, and this is known as Nurse Initiated and Managed Antiretroviral Therapy (NIMART).^4^ NIMART is credited for distinct benefits, such as earlier, quicker patient uptake with improved patient retention, enhanced patient outcomes, greater access to care (especially for those in rural communities) and reduced travel expenses to patients.^5^ The effectiveness of this programme depends on various factors as shown in other studies.

In a study executed in Uganda, the challenges experienced during the implementation of the PMTCT programme were investigated.^6^ The study concluded that there was a lack of infant testing and early diagnosis, patients’ non-adherence to medical instructions, staff shortages, inadequate facilities and equipment that impede the implementation of the programme.

In a study in Mozambique, it was found that the integration of the PMTCT programme, voluntary counselling and testing (VCT) and highly active antiretroviral therapy (HAART) services caused an increase in the duty burden of the nurses, resulting in a staff shortage. It was also found that the nurses lacked cultural sensitivity, which made patient education ineffectual – which in turn led to the impairment of the implementation of the various programmes.^7^

A third study in KwaZulu-Natal, South Africa, focused on the implementation of programmes implemented at 27 PHC sites (peri-urban and rural).^8^ It found that the PMTCT programme had shown significant improvements and achieved high coverage of interventions during pregnancy and delivery, but coverage of interventions for infants remained poor. Many PHC facilities had designated health workers for the PMTCT programme, but fragmentation of services created challenges with regard to patient access.

The fourth study was conducted in Eastern Cape and in Gauteng to evaluate health system weaknesses in accessing PMTCT services in South Africa.^9^ The study revealed weaknesses within the implementation of the programme such as shortages in staff, shortages of supplies, delayed HIV testing and treatment, delayed payment to lay HIV counsellors, delays in the receipt of laboratory results and the initiation of antiretroviral (ARV) therapy. A lack of healthcare worker education and fragmentation of services also caused delays in HIV diagnosis and care.^9^

It is clear from the abovementioned studies that existing information is outdated because of the changes that have taken place in the PMTCT guidelines, and does not reflect the quality of the programme from the perspective of nursing responsibilities.

## Objectives

The study’s main aim was to determine what the RNs’ perspectives are on the PMTCT programme as implemented at four PHC facilities in the Limpopo Province in order to discover whether any improvements could be suggested to guide management in its strategies to provide quality PMTCT healthcare service.

## Research design and methodology

This study followed the format of a qualitative, descriptive research design. The data were collected during semi-structured interviews using an interview guide. Open-ended questions were used, followed by more targeted open-ended probe questions.^10^

The study sites were purposively selected, and four PHC facilities in the Polokwane District, of the Limpopo Province, were chosen. These sites were chosen because they were easily accessible and considered safe to access. The four sites each serve the general population in Polokwane, as well as villages in the district and serve approximately 5000 patients per month (i.e. individual patients visited these PHC facilities for all the various services provided, not only for PMTCT services). The sites that were visited included three remote sites, varying in services that are available to the areas’ patients. Polokwane is the capital of the Limpopo Province and the selected facilities are typical PHC facilities in the district.

A pilot study was undertaken before implementing the final research. The interview guide was tested on a small sample of five participants and resulted in a few small adjustments to the interview protocols.

The sample for the study was purposively selected and participants were chosen based on the inclusion criteria.^11^ Twenty-one participants (*n* = 21) were identified and interviewed until data saturation was reached (Table 1). It was decided that data saturation occurred when the same responses were recorded 10 times. The sample size constituted approximately 40% of all the available registered nursing staff members across all four facilities. Included in the study were RNs with at least one years’ experience in PMTCT implementation, who agreed to participate in the study. The interviews were then conducted and data were collected in July 2014. The audio recordings with their accompanying field notes were transcribed, and the data from the interviews were analysed using thematic analysis.^12^

**TABLE 1:** The demographic data of participants (*n* = 21) in a qualitative study of the prevention of mother-to-child transmission programme in the Polokwane District.

Data	Frequency	%
**Gender**		
Female	18	86
Male	3	14
**Totals**	**21**	**100**
**Age range**		
20–29	3	14.2
30–29	1	5
40–49	5	23.8
50–59	8	38
60–69	4	19
**Total**	**21**	**100**

The trustworthiness and credibility of the research was accomplished by prolonged engagement, referential adequacy and member checks with the participants to ensure the correct interpretation. To ensure dependability and confirmability, a subject specialist from the Department of Health (DoH) and Social Development in Limpopo Province was consulted to audit the study. A clearance certificate (M131164) to conduct the study was obtained from the University of the Witwatersrand Human Research Ethics Committee (Medical), as well permission from the Limpopo DoH and Social Development, and the CEO of the Polokwane/Mankweng Hospital Complex. All participants provided written informed consent.

## Findings

### Demographic data

The demographical data of the participants are depicted in Table 1.

### To record and analyse the perspectives of the registered nurses on the successes on the implementation of the national prevention of mother-to-child transmission guidelines

The majority (*n* = 16) of the participants were of the opinion that the PMTCT programme is working effectively, and half (*n* = 8) of them thought that there is now an earlier antenatal-booking trend amongst patients. The term ‘early booking’ refers to when a patient first seeks healthcare during her pregnancy. HIV-positive women are then initiated on ART on the same day, regardless of their gestation or CD4 cell count. It is pertinent that the majority (*n* = 15) of the participants were of the opinion that there is a decreased rate in positive polymerase chain reaction (PCR) rates in infants at the time of the study. In Limpopo, a decline was shown in the mother-to-child transmission of HIV (MTCT) rates for the period 2013/14 where the transmission rates for infants (at 6–8 weeks) was 2.3%.^13^

Five of the participants (*n* = 5) viewed the education of the staff members as a contributing factor to the successes of PMTCT, as was their effective teamwork (*n* = 7) and positive attitude and commitment (*n* = 6). A substantial number (*n* = 19) of participants verbalised that they have updates on the PMTCT programme, as well as the PMTCT policies and guidelines, available to them. Other factors were also mentioned: nurses felt that they educated patients and that their effective communication with the patients and the DoH (monthly site visits from the DoH delegate) were key in the success of the PMTCT programme.

### To record and analyse the perspectives of the registered nurses on the challenges on the implementation of the national prevention of mother-to-child transmission programme guidelines

Four main themes under challenges were identified, namely: ‘Staffing’, ‘Late bookings’, ‘Patient education’ and ‘Resources’. Under ‘Staffing’, the main challenge that was experienced was staff shortages because of various reasons, such as a general nursing shortage (*n* = 7) and a time-consuming programme (assessing patients) (*n* = 5). Other challenges were that participants were not given advance notice of planned continuous education (*n* = 3). Even though a majority of participants (*n* = 19) were of the opinion that they received the necessary timely updates on the PMTCT guidelines, there were a few participants who felt that they did not receive regular continuous education from the DoH (*n* = 3).

The second theme under challenges was ‘Late bookings’. There were participants who felt that there are prevailing problems when it came to the timing of patients’ booking for antenatal care. Some of the participants felt that human migration contributed to a late booking tendency (*n* = 10), and others believed that patients’ cultural convictions (*n* = 7) prevented them from seeking care early in pregnancy. The participants also thought that teenagers (*n* = 6) were less likely to seek antenatal care early in pregnancy. The last contributing factor to late bookings that was identified by the participants as a lack of support systems (*n* = 5) and that patients who do not have a supportive family or community around them tend to book later for PMTCT care.

The third theme ‘Patient education’ indicated that there were challenges that could be linked to the patients’ misconception or misunderstanding of HIV, HIV treatment and the general intention of PMTCT. Some of the participants were of the opinion that patients still experienced a fear of being stigmatised (*n* = 6) by their communities and denied their HIV status. Two participants commented that patients did not adhere to the treatment that was prescribed and that patients experienced information overload (*n* = 2) when diagnosed. Participants also experienced language as a barrier (*n* = 2) when interacting with immigrant patients, and some of the participants felt that patients did not breastfeed exclusively, and mixed the feedings (*n* = 2). Other patient education issues discussed by the participants included the following: patients did not follow-up or were late in following up on testing of their infants at 18 months (*n* = 1) and that traditional remedies (*n* = 1) are still being used in conjunction with ART.

The fourth theme identified under challenges was ‘Resources’ and could be classified as facility management problems. A number of participants felt that there were general equipment shortages (*n* = 4). The nurses also felt that their facilities were in a state of disrepair (*n* = 3). Other challenges experienced were medication shortages (*n* = 2) and overdue blood results (*n* = 1).

### The third main finding identified is ‘Possible solutions’

There are four themes under ‘Possible solutions’. The first theme, ‘Staffing’ indicated that participants felt that the nursing shortage can be addressed by the recruitment of more staff members (*n* = 5). Advance notice of upcoming training (*n* = 1) and employing limited-period contract nurses (*n* = 1) in the PHC facilities in the event that permanent nurses are assigned for extended periods of training, also featured.

The second theme is ‘Education’. Participants (*n* = 8) thought that national campaigns using the media (radio, television and billboards) could address both late bookings and the lack of patient knowledge. Participants (*n* = 6) also felt that involving the communities – for example, during imbizos (or community meetings) – in educational campaigns could address the lack of knowledge on HIV and HIV treatment and improve the general understanding of the objectives of the PMTCT programme. In addition to these suggestions, the participants (*n* = 6) also felt that school education programmes could help solve the late booking tendencies amongst pregnant teenagers.

The third theme is ‘Immigration: Home Affairs and Stakeholders’. The participant who suggested this felt that the Department of Home Affairs could convene stakeholder meetings to address late bookings because of human migration (immigration), by educating immigrants on the importance and availability of antenatal care.

Theme four ‘Computer database’ was identified when a participant recommended that a national computer database could streamline appointments and facilitate patient follow-up regardless of where they might relocate. Other participants (*n* = 4) were of the opinion that the employment of tracing teams could help locate patients who do not follow-up with PMTCT care or could actually find pregnant women who are not seeking antenatal care.

## Discussion

This study found, according to the perceptions of the nurses working at the implementation level of the PMTCT guidelines, that the PMTCT programme is effective and that there is a definite tendency of pregnant women to report earlier for antenatal care. The PMTCT programme has made significant advances and is available at approximately 98% of the PHC facilities across the country. Importantly, according to the participants, fewer HIV-positive infants are born. With the steady increase in mothers taking part in the programme, the South African MTCT rates have dropped significantly to 1.3% in 2014.^3^ This supports the participants’ opinions. Nurses feel that their education and commitment is instrumental in the success of the PMTCT programme. The study found that an overwhelming number of the participants agreed that they had received the necessary timely education (*n* = 5), support manuals and guidelines from the DoH (*n* = 19) to implement the PMTCT programme. A few participants (*n* = 3) did, however, mention that they did not receive regular continuous education on the PMTCT programme from the DoH. This could suggest fragmentation of learning scheduling at either the DoH or the PHC facility level.

However, in spite of the efficacy of the programme, the participants were of the opinion that their patients and the communities they come from have a significant lack of knowledge where it pertains to HIV and HIV treatment. This lack of understanding prevented women from seeking care earlier in pregnancy and reduced their adherence to the prescribed treatment. Therefore, it was concluded that communities in the Limpopo Province still need more education on HIV, HIV treatment and the PMTCT programme.

**FIGURE 1:**
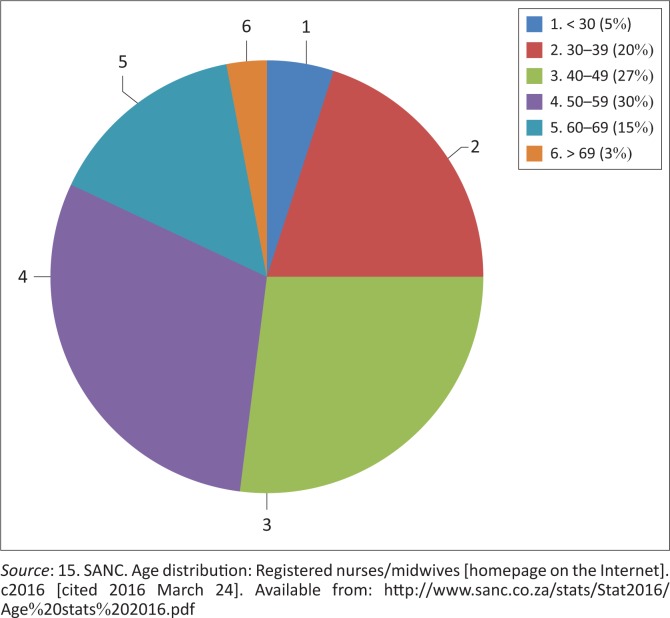
Age distribution of registered nurses in South Africa in December 2016.

With the increased burden of NIMART, nurses feel overwhelmed at times, having to spend a great deal more time per patient. There is an increased work burden, but no additional support is given. This staff shortage could lead to longer waiting periods for patients at PHC facilities and decreased morale amongst staff members. In this study, it was found that 19% of the nurses were already at the generally accepted retirement age, and 38% of the nurses will be at retirement age in under 10 years. According to SANC,^14^ 18% of RNs in South Africa are at retirement age (60 years to > 69 years), and 30% are close to retirement age (50 years to 59 years). More overwhelmingly, only 5% of RNs nationally are under the age of 30 years; that is, the cadre of younger nurses is alarmingly small (Figure 1).

It is clear that the nursing population at the sites studied is aging, and this could be a contributing factor to the nursing shortage currently experienced at these PHC facilities.

In this study, the participants stressed that the ineffective management of facilities, dilapidated buildings and shortages of working equipment could lead to protracted patient visits. According to Ho,^15^ this particular problem is also relevant in the Mpumalanga Province (Gert Sibande District) where buildings and facilities are inadequate, unhygienic and unsafe, resulting in substandard patient care. Rutter^16^ also referred to the mounting evidence of inefficiencies and undignified conditions patients in the Free State are subjected to. The findings of the People’s Commission of Inquiry into the Free State healthcare system, indicate that facilities are often in disrepair and equipment broken or unavailable. The facility management challenges seem to be a countrywide problem and do not only influence the implementation of the PMTCT programme but other healthcare programmes as well.

During the interviews, participants were asked how they would address the challenges that they experienced. A general idea that emerged from this study is that the communities around the Polokwane District needed more education and that this could be addressed by launching more national and community campaigns to increase adherence to the PMTCT programme and encourage earlier bookings. It was also found that the participants believed the nursing shortage could be addressed by staff recruitment and the implementation of support services like tracing teams.

### Limitations

Data were only collected at four PHC facilities in the Polokwane District of the Limpopo Province. Because of the small sample size, it will not be possible to generalise conclusions to all PHC facilities in the Limpopo Province, and these conclusions may not be applicable to all South African PHC facilities. The language barrier was partly addressed by the use of a fieldworker who was able to assist in the clarification of any questions and responses of the participants and the researcher. This was accomplished by communicating in the mother tongue of the participants. In some cases, however, it appeared that the participants comprehended the questions fully but, on analysing the responses, some doubt may be cast on this assumption. During data analysis, it became evident that the fieldworker had simplified some of the questions to the effect that the questions were not as open-ended as originally intended. This may have limited the participants’ responses.

### Managerial suggestions

Several suggestions flowed from this study:

- Research could be done on the intake of new nurse trainees who are supposed to supplement and eventually replace the aging nursing population in South Africa. This could render a better explanation for the current reasons for the nursing shortage in South Africa and possibly discover ways to encourage young school leavers to choose nursing as a career.
- Further research is suggested to ascertain workload (nurse–patient ratios) at PHC facilities in the Limpopo Province. It is unclear whether these ratios are monitored, which prompts the researcher to propose this recommendation. This will ensure that nurses are not overburdened and, where needed, that more staff appointments could be made. It may also be considered to develop individual staff members within the aegis of personal and professional practice to potentially balance the stress of perceived staff shortages.
- A continued, increased effort from the DoH should be made to increase the South African citizens’ knowledge on the HIV and PMTCT by initiating national, community and school campaigns.
- On a more practical and selective level, the researcher saw the need for more personalised patient education. Patients should be issued with a ‘take-home’ information booklet by the nurses. This booklet should contain general information on HIV (emphasising the importance of viewing HIV as a chronic medical problem), the patient’s treatment regimen how to correctly take medication, as well as how to improve health and well-being. This information booklet could be a useful tool in supporting and educating patients and their immediate family members and may combat information overload and confusion.
- Clinic facilities, that is, buildings and equipment should be improved. The sites visited were in a state of disrepair and needed improvement and regular maintenance (as mentioned by the participants). This could be addressed by training the facility managers, as well as conducting an audit of the infrastructure that could reveal the extent of the maintenance required to repair those buildings that are in disrepair. Another audit is recommended to highlight the essential equipment that should be acquired in order to reduce patient waiting times.
- Advance notice of training from the DoH should be ensured, and nurse managers at the sites should communicate effectively with personnel to ensure that the staff members are informed of the planned training dates. PMTCT training requires frequent updated training and this should feature in the in-service and development plan each facility compiles annually.
- The implementation of a national computer database is suggested to ensure a better monitoring system. This will eliminate the unnecessary repetition of tests and treatment of migrating patients, resulting in a reduction of costs and ensuring that there is an accurate audit trail on all known patients throughout South Africa. This will also ensure a reduction of ‘loss to follow-up’ because of the improved patient tracking and tracing. This will require a significant financial investment in a real-time computer documentation system, so that information is directly available to any NIMART practitioner at any given time.

## Conclusions

MTCT rates have dropped significantly to 1.3% in 2014.^2^ In spite of the successes of the PMTCT programme, considerable challenges still prevail; lack of patient education, poor facilities management and staff shortages could potentially influence the implementation of the PMTCT guidelines negatively. The findings and commentary in this study call for further research about the perspectives of nurses on the PMTCT policies and guidelines, as well as other national healthcare guidelines which could result in policy changes and improvements on a national level. National policy and guideline committees need to take cognisance of the suggestions made by the healthcare workers on the ground. This requires a commitment to change management in order to accept the views expressed by the nurses working at the proverbial coalface.

## References

[1] Bhuyan A, Jorgensen A, Sharma S Taking the pulse of policy: The policy implementation assessment tool. Washington, DC: Future Group, Health Policy Initiative, Task Order 1; 2010.

[2] Peteke M Dealing with HIV the smart way. Nursing update. The magazine for the caring profession. Denosa. 2015; p. 20–21.

[3] South Africa Department of Health National consolidated guidelines for the prevention of mother-to-child transmission of HIV (PMTCT) and the management of HIV in children, adolescent and adults. Pretoria: Government Printer; 2014.

[4] Cameron D, Gerber A, Mbatha M, Mutyabule J, Swart H Nurse initiation and maintenance of patients on antiretroviral therapy: Are nurses in primary care PHC facilities initiating ART after attending NIMART training? S Afr Med J. 2012;102(2):98–100. https://doi.org/10.7196/SAMJ.5195 22310442 10.7196/samj.5195

[5] Davies NECG, Homfray M, Venables EC Nurse and manager perceptions of nurse initiated and managed antiretroviral therapy (NIMART) implemented in South Africa: A qualitative Study. BMJ Open Access [serial on the Internet]. c2013 [cited 2015 May 7]. Available from: http://www.bmjopen.bmj.com 10.1136/bmjopen-2013-003840 PMC3831110 24240142

[6] Nuwagaba-Biribonwoha H, Mayon-White RT, Okong P, Carpenter LM Challenges faced by health workers in implementing the prevention of mother-to-child HIV transmission (PMTCT) programme in Uganda. J Public Health. 2007;29(3):269–274. https://doi.org/10.1093/pubmed/fdm025 10.1093/pubmed/fdm025 17538192

[7] Agadjanian V, Hayford SR PMTCT, HAART, and childbearing in Mozambique: An institutional perspective. SpringerLink [serial on the Internet]. c2009 [cited 2012 June 2]; 13(1):103–112. Available from: http://link.springer.com/article/10.1007/s10461-009-9535-0 10.1007/s10461-009-9535-0 PMC2836932 19326206

[8] Horwood C, Haskins L, Vermaak K, Phakathi S, Subbaye R, Doherty T Prevention of mother to child transmission of HIV (PMTCT) programme in KwaZulu-Natal, South Africa: An evaluation of PMTCT implementation and integration into routine maternal, child and women’s health services. Trop Med Int Health. 2010;15(9):992–999. https://doi.org/10.1111/j.1365-3156.2010.02576.x 20561313 10.1111/j.1365-3156.2010.02576.x

[9] Sprague C, Chersich MF, Black V Health system weaknesses constrain access to PMTCT and maternal HIV services in South Africa: A qualitative enquiry. AIDS Res Ther. 2011;8(10):1–9. https://doi.org/10.1186/1742-6405-8-10 21371301 10.1186/1742-6405-8-10 PMC3058008

[10] De Vos AS, Strydom H, Fouché CB, Delport CSL Research at grass roots for the social sciences and human service professionals. 3rd ed. Pretoria: Van Schaik Publishers; 2009.

[11] Babbie E, Mouton J, Vorster P, Prozesky B The practice of social research. 9th ed. Cape Town, South Africa: Oxford University Press; 2009.

[12] Braun V, Clarke V Using thematic analysis in psychology. Qual Res Psychol. 2006;3(2):77–101. https://doi.org/10.1191/1478088706qp063oa

[13] Limpopo Provincial AIDS Council Annual Progress Report 2014/15. Provincial Strategic Plan 2012–2016. Pretoria: Government Printer; 2016.

[14] SANC Age distribution: Registered nurses/midwives [homepage on the Internet]. c2016 [cited 2016 March 24]. Available from: http://www.sanc.co.za/stats/Stat2016/Age%20stats%202016.pdf

[15] Ho U A district in despair: A snapshot of the state of health in the Gert Sibande district is not a pretty picture. 10th ed. p. 50–52. Johannesburg: Treatment Action Campaign & SECTION27.

[16] Rutter L Free State province: Is there any hope left? 14th ed. p. 42–47. Johannesburg: Treatment Action Campaign & SECTION27.

