# Corrigendum: Prevention of mother-to-child transmission of HIV guidelines: Nurses’ views at four primary healthcare facilities in the Limpopo Province

**DOI:** 10.4102/sajhivmed.v18i1.821

**Published:** 2017-12-14

**Authors:** Barbara A Hanrahan, Adri Williams

**Affiliations:** 0 Department of Nursing Education, University of the Witwatersrand

In the version of this article initially published, the affiliations for Barbara A. Hanrahan and Adri Williams were incorrect. The correct affiliations for both authors is the Department of Nursing Education, University of the Witwatersrand, South Africa. The error has been corrected in the PDF version of the article. The author apologises for any inconvenience that this omission may have caused.

